# Biogenic metallic nanoparticles as enzyme mimicking agents

**DOI:** 10.3389/fchem.2023.1107619

**Published:** 2023-03-07

**Authors:** Khanyisile Ngcongco, Suresh Babu Naidu Krishna, Karen Pillay

**Affiliations:** ^1^ School of Life Sciences, University of KwaZulu-Natal, Durban, South Africa; ^2^ Department of Biomedical and Clinical Technology, Durban University of Technology, Durban, South Africa

**Keywords:** biogenic metallic nanoparticles, nanozymes, capping agents, toxicity, green synthesis

## Abstract

The use of biological systems such as plants, bacteria, and fungi for the synthesis of nanomaterials has emerged to fill the gap in the development of sustainable methods that are non-toxic, pollution-free, environmentally friendly, and economical for synthesizing nanomaterials with potential in biomedicine, biotechnology, environmental science, and engineering. Current research focuses on understanding the characteristics of biogenic nanoparticles as these will form the basis for the biosynthesis of nanoparticles with multiple functions due to the physicochemical properties they possess. This review briefly describes the intrinsic enzymatic mimetic activity of biogenic metallic nanoparticles, the cytotoxic effects of nanoparticles due to their physicochemical properties and the use of capping agents, molecules acting as reducing and stability agents and which aid to alleviate toxicity. The review also summarizes recent green synthetic strategies for metallic nanoparticles.

## 1 Introduction

Recent advances in nanotechnology have allowed researchers to develop devices with promising potential for use in a wide variety of applications in biomedicine, biotechnology, environmental science, and engineering (Bundschuh et al., 2018; Dan, 2020). Nanoparticles are the basic fundamental component in nanotechnology with sizes that range from 1 to 100 nm (Alavi and Karimi, 2018; Khan et al., 2019; Speranza, 2021). These structures offer major advantages due to their unique physicochemical properties such as their small sizes and diverse morphologies, large surface area to volume ratio, and in the case of metallic nanoparticles, their magnetization (Bundschuh et al., 2018). These physicochemical properties can be exploited for a broad spectrum of applications and present possible solutions to emerging global issues such as antimicrobial resistance, environmental pollution, and energy and food production (Ealias and Saravanakumar, 2017).

There is thus a need for more sustainable methods of synthesizing nanoparticles that are non-toxic, pollution free and more environmentally friendly when compared to the conventional chemical and physical methods for nanoparticle synthesis (Alavi and Karimi, 2018; Huynh et al., 2020; Bahrulolum et al., 2021; Ying et al., 2022). Recent studies focused on the use of biological organisms including plants, bacteria, yeast, fungi, lichens or algae to synthesize nanoparticles; in a method referred to as biological synthesis (Patil and Chandrasekaran, 2020; Nguyen et al., 2021; Ying et al., 2022). Proteins, enzymes, phenolic compounds, amines, alkaloids and pigments are some of the molecules in plants and microorganisms that can synthesize nanoparticles due to their reduction capability (Nadaroglu et al., 2017). The chemical and physical methods of synthesizing nanoparticles involve the use of reducing agents and stabilizing agents for the reduction of metal ions and to prevent agglomeration of the nanoparticles, however, these agents tend to be toxic to the environment and significantly contributes to nanoparticle toxicity which is highly unfavourable especially in the biomedical field (de Lima et al., 2012; Qu et al., 2019; Huynh et al., 2020; Nayak et al., 2021). In biological synthetic methods, biological organisms can produce biomolecules that act as reducing and stabilizing agents (Bahrulolum et al., 2021). These agents are not harmful to the environment, and maintain the stability of the synthesized nanoparticles thereby rendering them non-toxic (Nadaroglu et al., 2017).

Earlier reviews have highlighted the nanozyme activity of various types of nanoparticles (Ragg et al., 2015; Wu et al., 2019; Wang et al., 2020b; Ge et al., 2022). Some excellent review articles have also highlighted the biogenic strategies of metallic nanoparticles and advances in their role for biomedical application (Singh et al., 2021a; Nayak et al., 2021; Srivastava et al., 2021; Nayak et al., 2022). This mini review is different in that in provides an up to date overview of various biogenic strategies for metallic nanoparticle production, the role of biogenic synthesis as capping agents and up to date use of biogenic metallic nanoparticles as nanozymes.

## 2 Biogenic metallic nanoparticles

Many different biological organisms have been found to have an ability to synthesize a variety of metallic nanoparticles, with the most recent (2018–2022) studies presented in Supplementary Table S1 in the Supplementary Information. Although Supplementary Table S1 covers plants, bacteria, fungi and lichen as systems that can be used for metallic nanoparticle production, it should be noted that Bryophytes also have an inherent ability to produce metallic nanoparticles. To the best of our knowledge, bryophytes have not been used to produce metallic nanoparticles from 2018, and thus a review by Srivastava *et al.* (2021) gives a good overview of the bryophytes used for metallic nanoparticle production (Srivastava et al., 2021).

Plants are promising candidates for nanoparticle synthesis because they detox and reduce the accumulation of metals as they alter the chemical composition of metals making them non-toxic and thus producing nanoparticles as a by-product (Nadaroglu et al., 2017; Zhang et al., 2020). Plant extracts such as sugars, flavonoids, sapogenins, proteins, enzymes, tannins, phenolics, alkaloids, steroids, and organic acids, can be obtained from plant parts, such as leaves, stems, roots, fruit, bark, flowers, seeds and buds (Moodley et al., 2018; Yulizar et al., 2020). The extracts act as reducing agents which result in the production of nanoparticles. Recently, plant extracts from *Citrus sinensis*, *Lawsonia inermis*, *Artemisia haussknechtii*, *Cochlospermum gossypium* and *Juglans regia* have been reported for their use in nanoparticle synthesis (Alavi and Karimi, 2018; Kredy, 2018; Srivastava et al., 2019).

**FIGURE 1:**
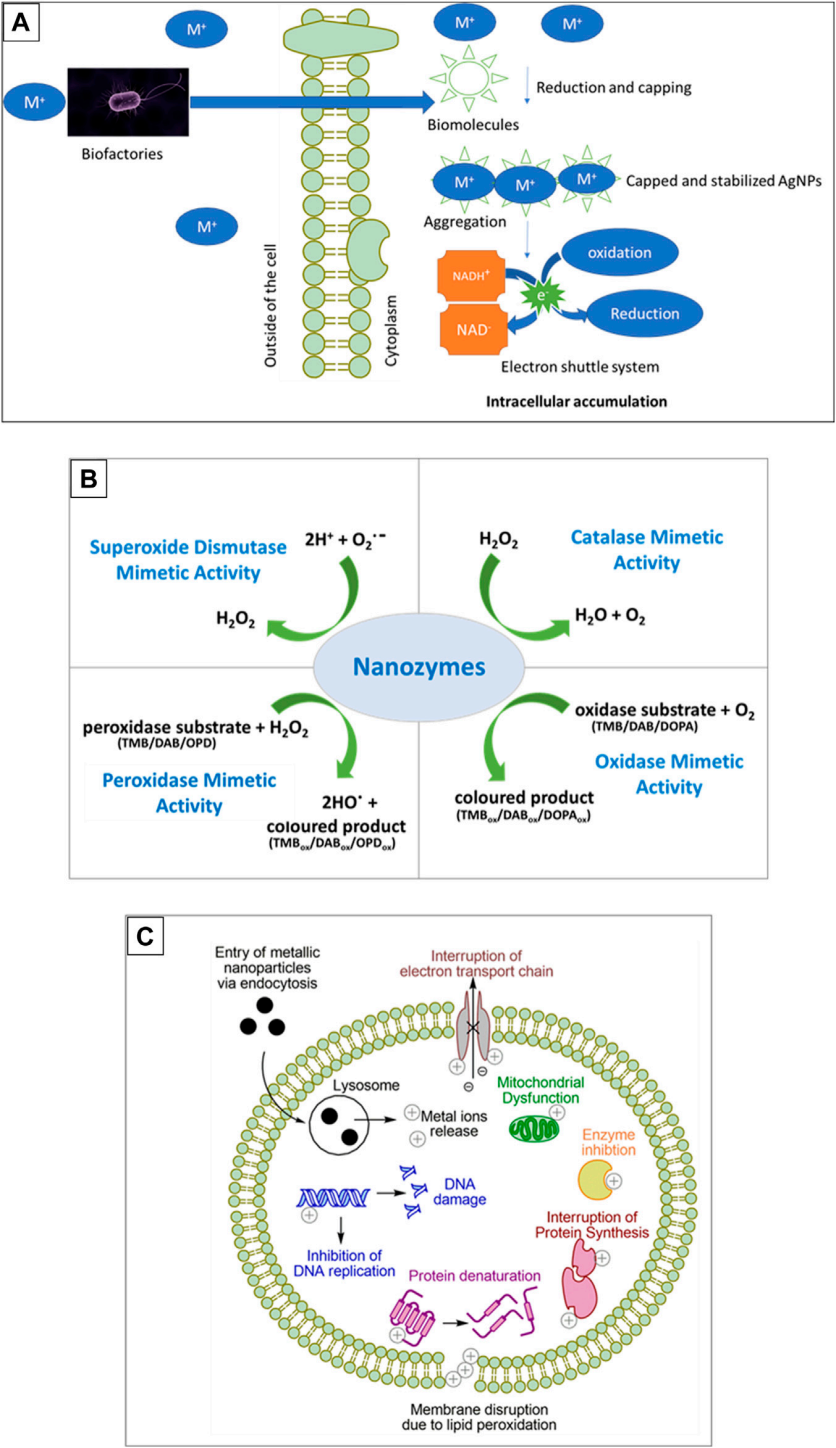
Synthesis, enzyme function and toxicity of metallic nanoparticles. **(A)** Is a schematic representation of biosynthesis of metal nanoparticles by microorganisms, **(B)** depicts the enzyme mimetic activity of biogenic nanoparticles and **(C)** shows the mechanisms of nanoparticle toxicity. Substrates used are 3, 3′, 5, 5′-Tetramethylbenzidine (TMB), 3, 3′-Diaminobenzidine (DAB), o-phenylenediamine (OPD), or 3,4-dihydroxyphenylalanine (DOPA).

Bacteria are also target candidates in nanoparticle production because of their rapid growth, cost-effectiveness, easy culturing, and since their growth conditions and environment can be easily controlled and manipulated (Nadaroglu et al., 2017). The emergence of resistance mechanisms in bacteria as a means of overcoming the harmful effects of metals also contributes to their ability to biosynthesize metallic nanoparticles. These mechanisms include transitions in the redox state, the operation of efflux systems, the buildup of metals inside the cell, intracellular precipitation, and extracellular creation of complexes (Figure 1A) (Moodley et al., 2021). These nanoparticles were believed to be formed through a method involving the NADH-dependent reductase enzyme, which goes through oxidation to create NAD^+^ and potentially, the lost free electron could turn Ag^+^ into AgNPs (Gurunathan et al., 2009; Sintubin et al., 2009).

Bacteria have the ability to convert heavy metal ions into nanoparticles by reducing them (Capuzzo, 2021). These advantages can therefore be exploited for nanoparticle synthesis. Bacteria including *Pseudomonas stutzeri*, *Desulfovibrio alaskensis*, *Morganella psychrotolerans* and *Lactobacillus casei* were recently reported to synthesize a variety of nanoparticles (Xu et al., 2018; Capeness et al., 2019).

Fungi are also ideal candidates for nanoparticle synthesis as their growth is easy and cost effective for laboratories and also at the industrial scale (Molnár et al., 2018). These organisms secrete a large number of enzymes and they have a large surface area due to their mycelia which play a vital role in rapidly forming nanoparticles as these characteristics causes metal precursor salts to be quickly converted to metallic nanoparticles (Khandel and Shahi, 2018; Li et al., 2021). Fungi such as *Ganoderma lucidum*, *Lignosus rhinocerotis*, *Trichoderma longibrachiatum*, and *Penicillium corylophilum* were recently used for the synthesis of metallic nanoparticles (Elamawi et al., 2018; Katas et al., 2019; Fouda et al., 2020; Nguyen et al., 2021).

## 3 The intrinsic enzyme mimetic activity of biogenic metallic nanoparticles

Nanoparticles are known to be multifunctional and among the functions that they possess is the ability to catalyse reactions. Initially, the catalytic activity of nanoparticles was a result of the conjugation of catalysts or enzymes to the shell of the nanoparticles and therefore the nanoparticles would provide magnetic properties while the catalyst or enzyme on the surface of the nanoparticles provided the catalytic activity (Gao et al., 2007). This drew the interest of researchers to other possible intrinsic enzyme-like activities that nanoparticles may possess.

Nanoparticles exhibiting enzyme-like catalytic activities, referred to as nanozymes, act as mimic enzymes that can replace natural enzymes because natural enzymes have disadvantages in their catalytic functions due to the high cost of production, the time consuming process for production, denaturation in harsh environmental conditions and therefore must have suitable pH and temperature, and specific substrates (Ragg et al., 2015; Ahmed et al., 2019; Singh, 2019; Rastogi et al., 2021). Since nanozymes are easy to produce with low cost, have high stability, and good robustness; they are suitable candidates for applications requiring catalytic functions and were found to possess enzymatic activity identical to that of peroxidase, haloperoxidase, oxidase, catalase, hydrolase, and superoxide dismutase as summarized in Figure 1B (Ragg et al., 2015; Ahmed et al., 2019). To date, there are more than 300 types of nanomaterials that have been found to possess the intrinsic enzyme-like activity (Gao and Yan, 2016).

Ferrihydrite nanoparticles synthesized from the bacteria *Comamonas testosteroni* exhibited peroxidase-like activity similar to that of horseradish peroxidase (HRP), and these nanoparticles were able to catalyze reactions of the peroxidase chromogenic agents 3, 3′, 5, 5′-Tetramethylbenzidine (TMB), 3, 3′-Diaminobenzidine (DAB), and o-phenylenediamine (OPD) in the presence of H_2_O_2_ (Ahmed et al., 2019). This peroxidase-like activity displayed by the bacteria was exploited to develop a colorimetric method for the detection of H_2_O_2_ and glucose which was used for successfully detecting glucose in human serum (Ahmed et al., 2019). Magnetic nanoparticles referred to as magnetosomes, synthesized from *Magnetospirillum gryphiswaldense* (magnetotactic bacteria) also exhibit intrinsic peroxidase-like activity indicated by their ability to catalyze TMB *in vitro* in the presence of H_2_O_2_ (Guo et al., 2012). The peroxidase-like activity of magnetosomes plays a role in reducing enhanced intracellular reactive oxygen species (ROS) levels generated under conditions having low oxygen and high iron concentration (Guo et al., 2012; Lin et al., 2019). ROS are very reactive chemical molecules containing oxygen, generated in cell organelles including the endoplasmic reticulum (ER), peroxisomes and the mitochondria (Yu et al., 2020b). This ROS elimination role is thought to be significant for the survival of magnetotactic bacteria growing under similar conditions (Guo et al., 2012; Lin et al., 2019). The peroxidase mimetic activity of magnetosomes has been used for the detection of H_2_O_2_ and glucose (Hu et al., 2010).

Plant extracts contain products that comprise functional groups including phenolic acids, proteins, polyphenol, bioactive alkaloids, terpenoids and sugars, which reduce metal ions in the synthetic mechanism for nanoparticles (Das et al., 2022a). These functional groups can stabilize the synthesized nanoparticles and improve their catalytic efficiency (Das et al., 2022a). Palladium nanoparticles synthesized using gum kondagogu, a tree gum from *C. gossypium*, were used for developing a colorimetric assay for quantifying cholesterol from human serum based on the peroxidase-like activity exhibited by the synthesized nanoparticles (Rastogi et al., 2021). This study showed the potential application of the intrinsic peroxidase mimicking properties of the palladium nanoparticles for diagnostic, detection and quantification purposes (Rastogi et al., 2021).

*Prunus nepalensis* fruit extract was used for synthesizing gold nanoparticles exhibiting peroxidase-like catalytic activity (Das et al., 2022a). The catalytic activity of the gold nanoparticles was confirmed by the ability to catalyze the oxidation of the substrate TMB in the presence of H_2_O_2_ (Das et al., 2022a). It was found that the gold nanoparticles exhibited a higher maximum reaction velocity and affinity for TMB compared to natural horse radish peroxidase (Das et al., 2022a). The improved catalytic efficiency of the gold nanoparticles is said to have been a result of the functional groups present in the fruit extract (Das et al., 2022a). This peroxidase-like activity of the gold nanoparticles was exploited for a potential colorimetric immuno-sensing assay for the detection of *Mycobacterium bovis*, a bovine tuberculosis transmitted from cattle to humans through the “consumption of unpasteurized milk” (Smith et al., 2004; Das et al., 2022a). Silver nanoparticles synthesized from *Cucumis sativus* and *Aloe vera* extracts, with the *A. vera* extract used as a capping agent, catalyzed the reduction of methyl orange dye and *para*-nitro-phenol (Riaz et al., 2022). This indicated the potential of these nanoparticles in the degradation of nitro-phenols that are found in industrial waste.

Ferrihydrite nanoparticles from *Trichoderma guizhouense* synthesized during interaction of the fungus with hematite, whereby fungi take up minerals to form nanoparticles, also exhibited peroxidase-like activity (Yu et al., 2020a). Fungi interact with minerals and biomineralize them into nanoparticles, and this interaction is known as fungus-mineral interaction. The interaction is important in the transformation of rocks, minerals and metals, the degradation of rhizospheric organic matter and phosphate fixation. The generation of ROS was observed during fungal growth and therefore, the concentration of these species must be maintained at sub-toxic levels (Yu et al., 2020a). The peroxidase-like activity of the synthesized ferrihydrite nanoparticles reduced the generated ROS, lowering their toxic effects. It was suggested that the production of the nanoparticles caused the ROS generated during fungal growth to decrease, therefore maintaining the concentration of the ROS at sub-toxic levels.

The enzymatic activity of biogenic nanoparticles similar to natural enzymes presents a platform whereby they can be exploited for developing several methods for diagnostic, detection and biosensing applications. To date, there are numerous patent applications and patents that have been granted for nanozyme production and nanozyme systems which highlights the importance of these small molecules. A list of these patents and patent applications are listed in Table 1, together with a summary of various applications of biogenic nanoparticles that have recently been evaluated, highlighting the versatility of these nano molecules.

**TABLE 1:** List of Nanozyme Patent Applications and Summary of Recent Nanoparticle applications.

Invention	Inventors	Application number	Year	Country	References
Nanozymes, methods of making nanozymes, and methods of using nanozymes	Cao, Y.C., Liu, C., Liu, H., Wang, Z., and YangS.H.	WO2011133504-A2	2011	WIPO (PCT)	Cao et al. (2011)
Synthesis method of enzyme-mimic magnetic nanocatalysts, and enzyme-mimic magnetic nanocatalysts thereby	Jang, J., Lee, S., and Lee, J	KR101350722B1	2014	South Korea	Jang et al. (2014)
Nanozymes, methods of making nanozymes, and methods of using nanozymes	Cao, Y.C., and Liu, C	WO2015023715A1	2015	WIPO (PCT)	Cao and Liu (2015)
Nanozymes, methods of making nanozymes, and methods of using nanozymes	Cao, Y.C., and Liu, C	US20160215279A1	2016	United States	Cao and Liu (2016)
Nanoparticle-attached enzyme cascades for accelerated multistep biocatalysis	Medintz, I.L., Vranish, J.N., Ancona, M., Susumu, K., and DiazS.A.	US20180171325A1	2017	United States	Medintz et al. (2017)
Stabilized polymeric nanocapsules, dispersions comprising the nanocapsules, and methods for the treatment of bacterial biofilms	Rotello, V.M., Landis, R.F., Gupta, A., and LeeY.W.	WO2017040024A1	2017	United States	Rotello et al. (2017)
PD-IR nanoparticles used as peroxidase mimics	Xia, X	20,170,199,179	2017	United States	Xia (2017)
Nanozymes, methods of making nanozymes, and methods of using nanozymes	Cao, Y.C., Liu, C., Liu, H., Wang, Z., and YangS.H.	US 10,081,542 B2	2018	United States	Cao et al. (2018)
Ig E detection and allergy diagnostic method using enzyme-mimetic nanozyme-based immunoassay	Lee, S., Lee, S., Kim, M., Shin, S., Lee, J., and Kim, M	WO2018084340A1	2018	WIPO (PCT)	Lee et al. (2018)
Enzyme-encapsulated nanoparticle platform	Ortac, I., Esener, S.C., Yayla, I.G., and Messmer, B	US10300152B2	2019	United States	Ortac et al. (2019)
RNA silencing nanozymes	Cao, Y.C., and Jiang, T	20210139873	2021	United States	Cao and Jiang (2020)

Application	**Function**	**References**			
Antimicrobial Agents	Activity against bacterial and fungal growth	Dong et al. (2019), Akpinar et al. (2021), Sarwer et al. (2022), Soliman et al. (2022)					
Bioremediation	Degradation of heavy metals, pesticides, insecticides, herbicides and dyes from polluted environments	Tripathi et al. (2018), López-Miranda et al. (2019), Chaudhari et al. (2022), Gami et al. (2022), Salama et al. (2022)					
Delivery Systems	Delivery of cancer targeting genes and therapeutic drugs	Abolhasani Zadeh et al. (2022)					
Enzyme Immobilization	Immobilization of enzymes from reaction mixtures	Fotiadou et al. (2021)					
MRI Contrast Agents	Molecular imaging, diagnosis and treatment of diseases	Cai et al. (2019), Nan et al. (2021), Wei et al. (2021)					

## 4 Toxicity of nanoparticles and the role of capping agents

Nanoparticle toxicity presents a challenge, especially in areas such as biomedicine, cosmetics, agriculture and the food science, and thus there is a need for the development of nanoparticles with low cytotoxicity. The main physicochemical properties that determine toxicity of nanoparticles are size, shape, surface chemistry, surface composition, surface area to volume ratio and stability (Aillon et al., 2009; Sukhanova et al., 2018). The primary mechanisms of toxicity of metallic nanoparticles stem from them entering the cell *via* endocytosis, which places them within an acidic lysosome resulting in oxidation and release of metal ions. The intracellular metal ions can then exert a myriad of toxic effects as summarised in Figure 1C (Repetto et al., 2010; Carrillo-Carrion et al., 2014).

Fortunately, encapsulation strategies have recently been on the radar of researchers in a bid to reduce the toxic effects of nanoparticles as it could provide stability to the nanoparticle and reduce its susceptibility to release metal ions.

Capping agents are molecules that play an essential role in the growth, aggregation, and physicochemical properties of nanoparticles by regulating their size, shape, geometry, and surface chemistry (Javed et al., 2022; Sidhu et al., 2022). Capping agents can firmly adsorb on the nanoparticle surface forming a single or multilayer protective coating thus providing long term nanoparticle stability (Javed et al., 2022). Capping agents can consist of proteins, carbohydrates, amino acids, and lipids (Bulgarini et al., 2021) and can prevent aggregation of nanoparticles (Sidhu et al., 2022).

Capping agents are found in biological organisms, and they can act as reducing and stability agents, an advantage that is noteworthy for biogenic nanoparticles (Guilger-Casagrande et al., 2021), (Javed et al., 2022). In addition, capping agents from biological organisms can be introduced to the chemical synthetic strategies and this has become an essential strategy in lowering the cytotoxic effects of chemically synthesized nanoparticles (Sidhu et al., 2022).

The encapsulation of nanoparticles with capping agents forms a barrier between the inner core of the nanoparticle and the surrounding environment, improving nanoparticle solubility, reactivity, interactions with biomolecules, preventing aggregation and inducing their biological functions (Weingart et al., 2013). A study by Guilger-Casagrande *et al.* (2021) evaluated the effects and functions of capping agents on the stability of silver nanoparticles synthesized by the fungal strain *Trichoderma harzianum* by comparing the stability of the nanoparticles with and without capping agents. It was found that when capping agents were removed from nanoparticles, the diameter of the nanoparticles increased and it was proposed that the reason for this may be “subsequent aggregation of the nanoparticles” (Guilger-Casagrande et al., 2021).

Soliman *et al.* (2022) suggested that extracellular enzymes and proteins from *Trichoderma saturnisporum* acted as capping agents in the synthesis of silver and gold nanoparticles. Puri and Patil (2022) confirmed the presence of phytochemicals by screening *Tinospora cordifolia* extract used for synthesizing selenium nanoparticles. Among the phytochemicals present in the extract were phenolics and flavonoids and the hydroxyl groups of these biomolecules were proposed to act as capping agents (Puri and Patil, 2022). Highly stable, negatively charged, spherically shaped and nano-sized selenium nanoparticles were synthesized from the plant extracts and it was suggested that the stability of the nanoparticles was due to the phytochemicals detected in the extract (Puri and Patil, 2022). Silver nanoparticles synthesized using yeast extract were capped by biomolecules from the extract, and the resulting silver nanoparticles exhibited shapes, sizes and surface chemistry that exhibited good long-term stability (Shu et al., 2020).

Biogenic nanoparticles thus present a promising potential for a variety of applications with the added advantage of low toxicity due to their inherent capping potential, a feature that is lacking in physicochemical synthetic strategies that are reliant on addition of synthetic capping agents resulting in labor intensive multi-step processes (de Lima et al., 2012).

## 5 Current scenarios and challenges

The emergence of nanotechnology has seen nanoparticles having widespread application and being the molecule of choice, resulting in them being in high demand. Although there are numerous synthetic strategies for nanoparticle production, the physical and chemical routes pose many challenges such as the need for expensive equipment, capping agents and harmful chemicals, and production of monodisperse nanoparticles with similar morphology is not so straight forward. Green synthetic methods, using inherent biological machinery and phytochemicals as capping and stabilization agents, is therefore seen as the preferred method for nanoparticle production. However, large scale production is still seen as a challenge and researchers are daily evaluating new strategies for scaling up. One of the novel methods that could be explored for large scale production of nanoparticles is investigating the role of soil microbes in influencing plant growth and uptake of nutrients. This could have importance in nanoparticle production as soil microbes could directly affect how metals and nutrients are taken up by plants before being packaged into nanoparticles (Das et al., 2022b).

## 6 Conclusion and future prospects

The use of biological organisms for synthesizing nanoparticles is increasing as these organisms produce their own reducing and stabilizing agents that are involved in reducing metal ions to nanoparticles and also providing encapsulation. The biogenic nanoparticles thus have the important benefit of reduced toxicity and have immense application in a wide variety of biotechnology and biomedical fields such as drug delivery systems, scanning techniques, cosmetics, and assay systems. There is thus huge potential for exploiting biogenic nanoparticles for our advancement, however the scaling up of nanoparticle production is still an area that requires more research. An exciting area of research that could possibly result in an effective scaling up mechanism could be the role of soil microbes in plant growth as a symbiotic relationship could potentially be manipulated to allow improved metal and nutrient uptake by the plants thereby resulting in increased nanoparticle production.
