# Supplementary material for: Biogenic metallic nanoparticles as enzyme mimicking agents
Source: Front Chem. 2023 Mar 7;11:1107619. doi: 10.3389/fchem.2023.1107619 (PMC10027806; doi:10.3389/fchem.2023.1107619)
Supplement: Supplementary file 1 [file Table1.docx]

Supplementary Information

Table S1: Summary of metallic nanoparticles synthesized by biological organisms (2018-2022)

| Organism | Source | Nanoparticle compositions | | References |
| --- | --- | --- | --- | --- |
| Plants | *Nepeta deflersiana* | Ag | | (Al-Sheddi *et al.*, 2018) |
|  | *Artemisia haussknechtii* | Ag, Cu, and Ti | | (Alavi and Karimi, 2018) |
|  | *Elettaria cardamomum* | MnFe_2_O_3_ | | (Chaudhari *et al.*, 2022) |
|  | *Juglia regia* | Ag | | (Khorrami *et al.*, 2018) |
|  | *Sargassum wightii* | Mg | | (Pugazhendhi *et al.*, 2019) |
|  | *Citrus sinensis* | Ag | | (Srivastava *et al.*, 2019) |
|  | *Artemisia annua* | Zn | | (Wang *et al.*, 2020a) |
|  | *Cochlospermum gossypium* | | Pd | (Rastogi *et al.*, 2021) |
|  | *Annona squamosa* | | Cu | (Singh *et al.*, 2021b) |
|  | *Cucumis sativas* | | Ag | (Riaz *et al.*, 2022) |
|  | *Aloe vera* | | Ag | (Riaz *et al.*, 2022) |
|  | *Lawsonia inermis* | | Ag | (Kredy, 2018) |
|  | *Leucophyllum frutescens* | | Ag and Au | (Gami *et al.*, 2022) |
|  | *Zingiber officinale* | | MnFe_2_O_3_ | (Chaudhari *et al.*, 2022) |
| Bacteria | *Lactobacillus casei* | | Se | (Xu *et al.*, 2018) |
|  | *Acinetobacter sp.* | | Ag | (Nadhe *et al.*, 2019) |
|  | *Comamonas testosteroni* | | Fe_3_O_4_ | (Ahmed *et al.*, 2019) |
|  | *Desulfovibrio alaskensis* | | Pt, Pd, and Pd/Pt | (Capeness *et al.*, 2019) |
|  | *Klebsiella pneumonia* | | Ag | (Saleh and Alwan, 2020) |
| Fungi | *Trichoderma longibrachiatum* | | Ag | (Elamawi *et al.*, 2018) |
|  | *Ganoderma lucidum* | | Ag | (Nguyen *et al.*, 2021) |
|  | *Penicillium verrucosum* | | Ag | (Yassin *et al.*, 2021) |
|  | *Trichoderma harzianum* | | Ag | (Guilger-Casagrande *et al.*, 2021) |
| Lichen | *Usnea longissima* | | Ag | (Siddiqi *et al.*, 2018) |
|  | *Cetraria islandica (L.) Ach.* | | Ag, Au and Ag-Au | (Ciplak *et al.*, 2018) |
|  | *Protoparmeliopsis muralis* | | Ag, Cu, Ti, Zn and Fe | (Alavi *et al.*, 2019) |
|  | *Parmelia sulcata* | | Au | (Gandhi *et al.*, 2019) |
|  | *Lecanora muralis* | | Zn, Ti, Si and Fe | (Abdullah *et al.*, 2020) |
|  | *Parmelia sulcata* | | Ag | (Gandhi *et al.*, 2021) |
